# Effects of education, income, and occupation on prevalence and symptoms of knee osteoarthritis

**DOI:** 10.1038/s41598-021-93394-3

**Published:** 2021-07-07

**Authors:** Ji Yeon Lee, Kyungdo Han, Yong Gyu Park, Sung-Hwan Park

**Affiliations:** 1 Division of Rheumatology, Department of Medicine, The Catholic University of Korea, Seoul St. Mary’s Hospital, Seoul, South Korea; 2 Department of Biostatistics, The Catholic University of Korea, Seoul, South Korea

**Keywords:** Musculoskeletal system, Risk factors, Osteoarthritis

## Abstract

To examine the effect of socioeconomic status (SES) as measured by three components of education level, income level, and occupation on prevalence and symptom severity of knee osteoarthritis (OA) and to determine which of these factors has the strongest association. We conducted a cross-sectional study using data from the Fifth Korean National Health and Nutrition Examination Survey that were collected between 2010 and 2012. Male and female participants 50 years or older were included. Analyses to examine the associations of the three SES components with prevalence and symptom severity of knee OA were performed. A total 9,071 participants was included in the study. As expected, lower education, lower income level, and non-managerial or no job were associated with higher prevalence of knee OA and knee symptoms. Among the three SES components, lower education was most strongly associated with knee pain and radiographic knee OA after adjusting for the other two. Lower education level is the component of SES that most strongly relates to higher prevalence of knee OA and knee symptoms. Improving societal education level might decrease the socioeconomic burden of knee OA.

## Introduction

Osteoarthritis (OA) is the most common form of arthritis worldwide, and the many established risk factors include systemic factors and local factors such as age, sex, obesity, genetics, and injury^1,2^. Low socioeconomic status (SES) also is a risk factor of OA, the main components of which used to determine SES are income, education, and occupation. Previous studies have reported that low educational attainment was associated with higher prevalence of OA^3^ and pain^4,5^. Occupation also was associated with symptomatic radiographic knee OA^6^. Low education, non-managerial occupation, and poverty status all predicted pain, physical dysfunction, and disability among adults with OA^5,7,8^. Although these associations could be attributed to a greater amount of strenuous physical activity among people with low SES, previous studies have demonstrated that low SES itself is related to prevalence and symptoms of OA after adjusting for possible confounders such as age, sex, body mass index(BMI), injury, and smoking^9–11^.

SES is a composite measure of a person's work experience and of an individual's or family's economic and social position in relation to others. Typically, household income, earner education, and earner occupation are examined to assess SES. In some instances, however, the three components are not directly proportional. For example, one might have attained a high level of education but is employed in a non-managerial job with low compensation. Another can have a high-compensation job but was poorly educated. Although all three factors have been reported to be associated with knee OA, it is not known which has a greater impact on prevalence or severity of the disease. By knowing the separated contribution of SES, clinicians can better educate and focus on the at-risk groups which need earlier monitoring and prevention for OA. It is important especially given the fact that the disease has no definite treatment so far.

Therefore, in this study, we used data from a Korean nationwide survey to examine the relationship of socioeconomic status as measured by its three dimensions of education level, income level, and occupation to prevalence and symptom severity of knee OA after adjusting for possible confounders including age, sex, BMI and SES factors. Among the three dimensions, we examined which has a greater impact on prevalence of knee OA and knee symptoms.

## Results

### Study participants

A total of 9,071 participants, 5,193 (57.2%) women and 3,878 (42.8%) men, was included in the study. Of these, 36.3% had radiographic knee OA. Radiographic knee OA was more prevalent in women: 44.8% of female participants had radiographic knee OA, whereas 24.9% of male participants had radiographic knee OA. Among those with radiographic knee OA, 70.7% were women. Among those without radiographic knee OA, the proportions of male and female participants were equivalent.

Table 1 shows the characteristics of participants by sex and by presence of radiographic knee OA (Kellgren-Lawrence (KL) grade $\ge$ 2). In both men and women, those with radiographic knee OA were significantly older, more obese, less likely to be smokers, and more likely to have symptoms of knee pain or stiffness compared with those without radiographic knee OA. Men and women with radiographic knee OA also were more likely to have radiographic spondylosis. Interestingly, women with radiographic knee OA were less likely to be married compared with those without radiographic knee OA, but this did not apply to men. As expected, living in an urban area, higher education status, higher income level, and a managerial occupation were related to lower prevalence of radiographic knee OA.

**Table 1:** Table 1. Subject characteristics.

	Male			Female		
	RKOA(-)	RKOA( +)	P-value	RKOA(-)	RKOA( +)	P-value
	N = 2913	N = 965		N = 2867	N = 2326	
Age, yrs	59.3 $\pm$ 0.2	67.8 $\pm$ 0.3	< .0001	60.1 $\pm$ 0.2	66.2 $\pm$ 0.4	< .0001
BMI, kg/m^2^	23.7 $\pm$ 0.1	25 $\pm$ 0.1	< .0002	23.7 $\pm$ 0.1	24.3 $\pm$ 0.1	< .0002
Waist circumference, cm	80.4 $\pm$ 0.2	84.6 $\pm$ 0.3	< .0003	84.8 $\pm$ 0.2	86.8 $\pm$ 0.4	< .0003
Current smoker, %	34.9 (1.2)	27.8 (1.8)	0.0014	5.1 (0.6)	3.4 (0.5)	0.0371
EtOH use, %	33.6 (1.2)	32.8 (2.1)	0.7506	2.3 (0.4)	1.5 (0.3)	0.1065
Regular exercise, %	21 (1)	20.8 (1.7)	0.9072	16.4 (0.9)	14.1 (0.9)	0.0591
Urban living, %	73.9 (2.2)	67.6 (3.2)	0.0085	77.2 (2.1)	64.8 (2.8)	< .0001
Married, %	92.2 (0.7)	91.5 (1.2)	0.573	76.4 (1.1)	57.5 (1.4)	< .0001
Education attainment			< .0001			< .0001
Elementary	28.2 (1.2)	41.3 (2)		48 (1.2)	74.2 (1.2)	
Middle school	20.6 (1)	20.9 (1.7)		20.2 (0.9)	13 (0.9)	
High school	32.2 (1.2)	26.6 (1.7)		24.3 (1)	10.1 (0.8)	
College	19 (1)	11.2 (1.5)		7.4 (0.7)	2.8 (0.4)	
Income			< .0001			< .0001
Q1	21.4 (1)	34.6 (2.1)		22.9 (1)	45.7 (1.3)	
Q2	25.2 (1)	25.8 (1.9)		28.1 (1)	23.9 (1.1)	
Q3	24.5 (1)	21.2 (1.9)		23.4 (1)	16.8 (1)	
Q4	28.9 (1.1)	18.4 (1.8)		25.6 (1.1)	13.5 (0.9)	
Occupation			< .0001			< .0001
Managerial	27.0 (1.1)	13.0 (1.4)		23.1 (1.1)	11.7 (0.9)	
Non-managerial	45.8 (1.4)	49.7 (2.1)		26.1 (1.2)	27.0 (1.5)	
No job	27.2 (1.1)	37.3 (2.0)		50.8 (1.1)	61.3 (1.4)	
Knee pain, %	8.7 (0.6)	20.9 (1.5)	< .0001	16.9 (0.9)	44.5 (1.3)	< .0001
Knee stiffness, %	3.8 (0.5)	9.9 (1.2)	< .0001	8.3 (0.6)	27.2 (1.3)	< .0001
Radiographic spondylosis, %	22.6 (1.0)	38.8 (2.1)	< .0001	24.4 (1.0)	46.5 (1.4)	< .0001

Numbers are Mean $\pm$ SE or Mean% (SE).

BMI, body mass index; EtOH, ethanol; RKOA, radiographic knee osteoarthritis.

When income status was divided into four quartiles in both men and women, those in the lowest quartile were more likely to have radiographic knee OA, knee pain, and knee stiffness, and those in the highest quartile were less likely to have radiographic knee OA and to have less pain or stiffness. Among both men and women with radiographic knee OA, those in the lowest quartile of income level were more likely to have knee pain, stiffness, and radiographic spondylosis (Table 2).

**Table 2:** Prevalence of knee OA and knee symptoms by components of socioeconomic status.

	Income					Education					Occupation			
	Q1	Q2	Q3	Q4	*P *value	Elementary	Middle school	High school	College or more	*P *value	Managerial	Non-managerial	None	*P *value
**Male**														
Knee OA	30.3 (1.7)	21.6 (1.6)	18.9 (1.7)	14.6 (1.5)	< .0001	28.2 (1.6)	21.4 (1.8)	18.2 (1.3)	13.6 (1.7)	< .0001	11.5 (1.3)	22.7 (1.2)	27 (1.5)	< .0001
Knee pain	17.4 (1.3)	12.5 (1.3)	8.7 (1.2)	6.8 (1)	< .0001	9.2 (1)	6.3 (1)	2.5 (0.6)	2.6 (0.7)	< .0001	5.1 (0.9)	13.1 (1)	13.4 (1.1)	< .0001
Knee stiffness	17.5 (1.2)	13.6 (1.5)	6.5 (0.9)	5.8 (1)	< .0001	8.7 (0.9)	5.3 (0.9)	3.1 (0.7)	1.9 (0.6)	< .0001	1.8 (0.7)	5.8 (0.7)	6.8 (0.8)	< .0001
**RKOA**														
w/ knee pain	28.7 (2.5)	22.8 (3.1)	13.7 (3)	12.1 (3.4)	0.0005	28.4 (2.7)	23.3 (3.5)	12.8 (2.8)	8.3 (2.7)	0.0123	5.9 (2.2)	21.9 (2.2)	24.9 (2.4)	< .0001
w/ knee stiffness	14 (2.1)	11 (2.7)	4.4 (1.7)	6.7 (2.2)	< .0001	12.8 (1.9)	12.7 (2.7)	4.9 (1.9)	5.6 (2.4)	0.0125	2.2 (1.3)	10.8 (1.7)	11.3 (2)	0.0143
w/ spondylosis	49.7 (3.3)	35.1 (3.4)	38.1 (4.8)	24.1 (4)	< .0001	45.8 (3.3)	34.1 (4.4)	33.9 (3.6)	33 (5.6)	0.0298	19 (4.6)	36.9 (2.8)	48.2 (3.3)	< .0001
**Female**														
Knee OA	60.1 (1.4)	39 (1.6)	35 (1.9)	28.5 (1.8)	< .0001	53.7 (1.2)	32.5 (1.9)	23.7 (1.6)	22.3 (3.2)	< .0001	27.5 (2)	43.8 (1.7)	47.6 (1.1)	< .0001
Knee pain	42.2 (1.4)	26.6 (1.5)	20.3 (1.6)	18.3 (1.4)	< .0001	26.4 (1.4)	13.9 (1.2)	11 (1.2)	9 (1)	< .0001	19.2 (1.7)	29.3 (1.5)	31.6 (1.1)	< .0001
Knee stiffness	37.7 (1.1)	19.3 (1.7)	14 (1.3)	9.5 (1.7)	< .0001	22.5 (1)	9.2 (1.2)	6.9 (0.9)	4.2 (1.2)	< .0001	9.4 (1.3)	16.5 (1.3)	18.6 (0.9)	< .0001
**RKOA**														
w/ knee pain	52.7 (1.9)	42.1 (2.6)	33.2 (3.1)	34.9 (3.2)	< .0001	51 (1.5)	31.1 (3.2)	21.9 (3.4)	14.3 (4.6)	< .0001	35.7 (3.7)	42.1 (2.4)	47.2 (1.7)	0.0092
w/ knee stiffness	33.3 (2)	25.5 (2.5)	19.9 (2.6)	18.9 (2.7)	< .0001	31.4 (1.6)	17.6 (2.7)	13.9 (2.7)	8.9 (4.2)	< .0001	22.1 (3.4)	25.5 (2.3)	28.9 (1.6)	0.1411
w/ spondylosis	53.8 (1.9)	43.1 (2.6)	37.8 (3.1)	38.5 (3.3)	< .0001	52.4 (1.7)	34.5 (3.3)	25.4 (3.1)	21.5 (5.9)	< .0001	32.6 (4)	47.2 (2.7)	48.8 (1.7)	0.0009

Numbers are n% (SE).

OA, osteoarthritis; RKOA, radiographic knee osteoarthritis.

When education level was divided into 4 categories, participants with the lowest education level were more likely to have radiographic knee OA, knee pain or stiffness, and symptomatic knee OA as well as radiographic spondylosis in both men and women (Table 2).

Occupation was strongly related to prevalence of knee OA and knee symptoms. Those in managerial work, including office work and service jobs, had the lowest prevalence of radiographic knee OA and knee symptoms. Interestingly, those without a job had the highest prevalence of radiographic knee OA with knee symptoms and spondylosis in both men and women (Table 2).

**Figure 1:**
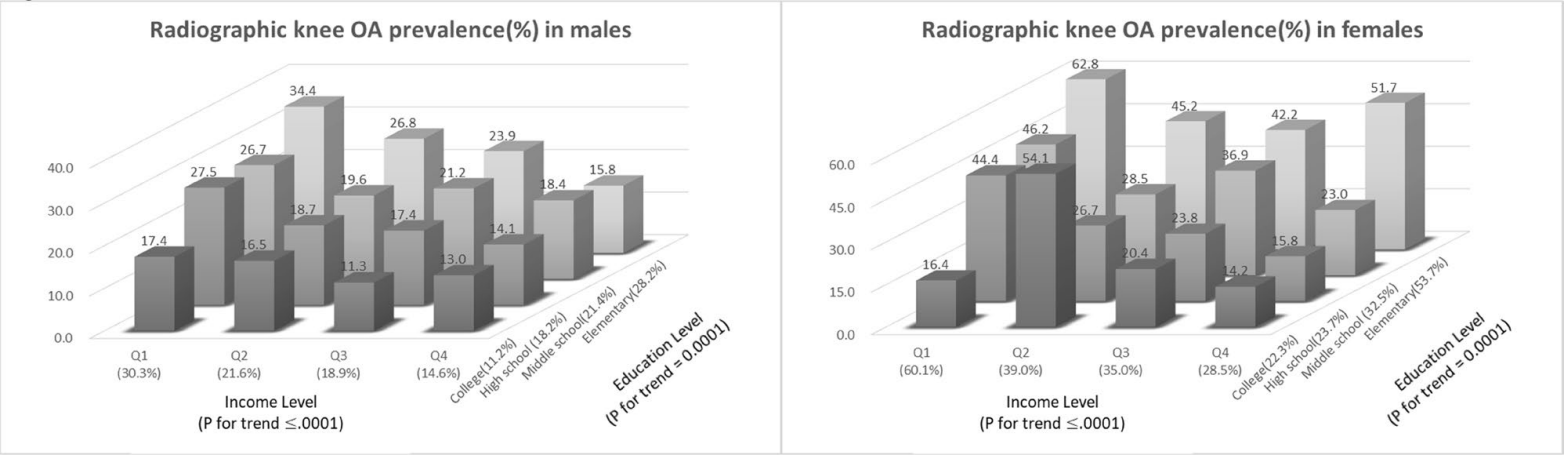
The prevalence of knee osteoarthritis (OA) according to income level and education status.

Figure 1 shows the prevalence of radiographic knee OA according to combined levels of income and education in men and women. Participants with the lowest income level and the lowest level of education had the highest prevalence of radiographic knee OA (34.4%), whereas those with highest education and income levels had the lowest prevalence of radiographic knee OA (11.3%). Notably, this pattern was less remarkable in female participants. Although those with the lowest income and lowest education showed the highest prevalence of knee OA (62.8%), those with the highest income level with low education status and those with highest education status with the second to lowest income level also had a high prevalence of radiographic knee OA (51.7% and 54.1%, respectively).

**Figure 2:**
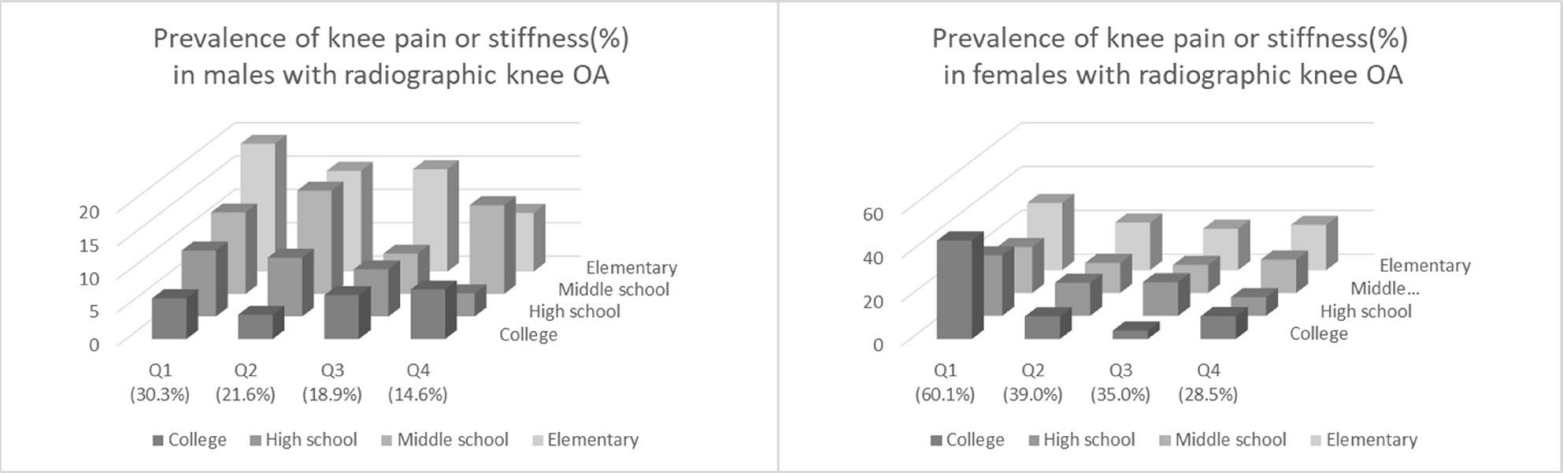
The prevalence of knee symptoms among those with radiographic knee osteoarthritis(OA) according to income level and education status.

When we ran the model with only participants with radiographic knee OA, knee symptoms were more prevalent in those with lower educational status in men and lower income level in women (Fig. 2).

### Severity of radiographic knee OA according to income, education, and occupation

**Figure 3:**
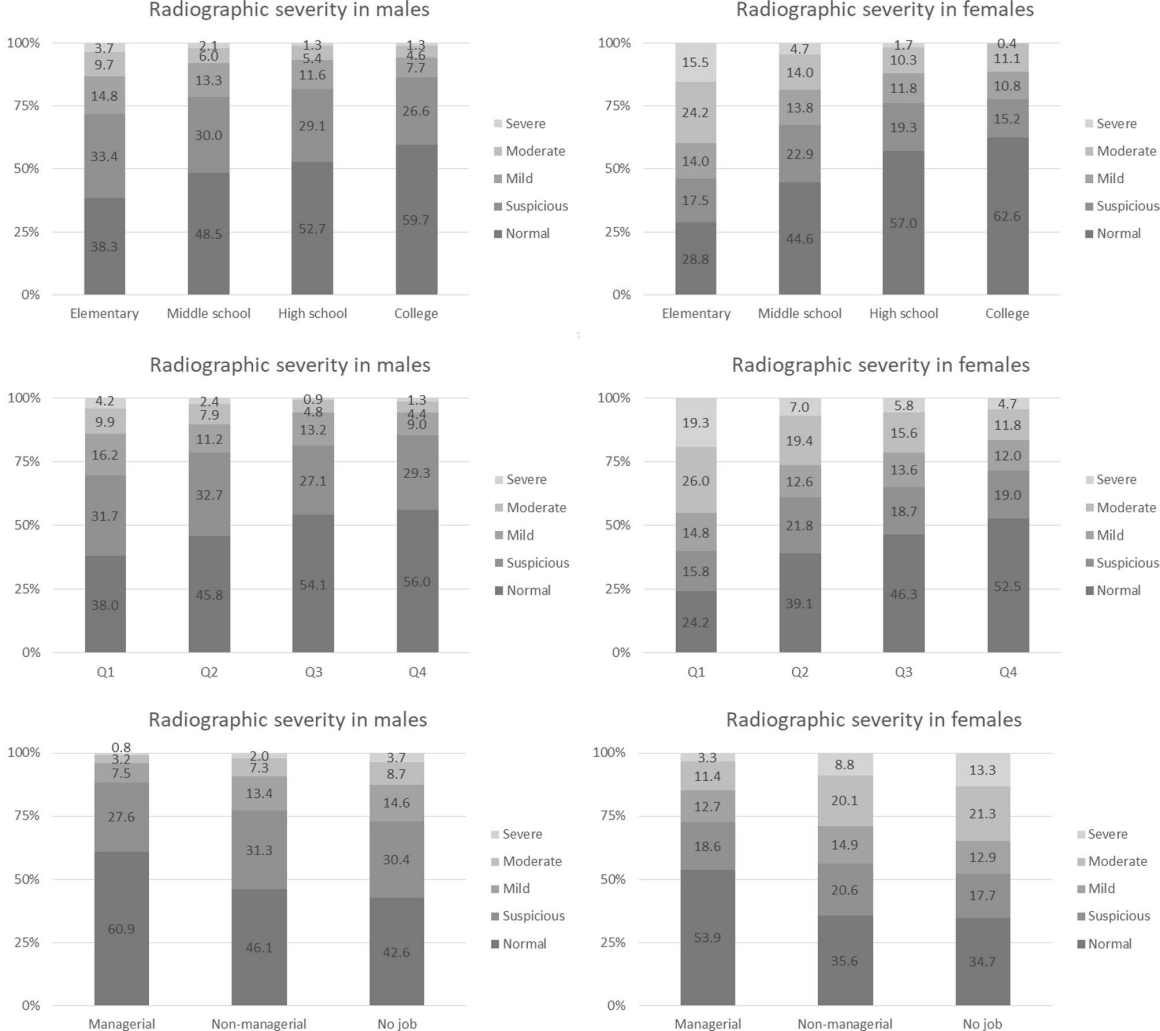
The radiographic severity of knee osteoarthritis (OA) according to income level, educational status, and occupation.

Figure 3 shows the radiographic severity of knee OA according to the levels of the three determinants of SES. Males or females who earned less income significantly were more likely to have severe radiographic knee OA, and this pattern was more evident in females. Of females in the lowest income quantile (Q1), 45.3% had moderate-to-severe knee OA, and this was evident according to education level and occupation. The more educated participants had milder radiographic knee OA compared to the less educated who had more severe radiographic knee OA. Of females who were educated through elementary school, almost 40% had moderate-to-severe knee OA. Regarding occupation, unemployed participants or those with non-managerial jobs had more severe knee OA. In women, 13% without a job had severe knee OA, while 3% with managerial jobs had severe knee OA. In men, 3.7% without a job had severe knee OA, while 0.8% with managerial jobs had severe knee OA.

### Comparison of the effects of the three socioeconomic factors on prevalence of radiographic knee OA

Table 3 shows the compared effects of education, income, and occupation on prevalence of radiographic knee OA and knee symptoms. All the models were adjusted for age, body mass index (BMI, kg/m^2^), and the other two of the three SES components. In the adjusted models, although subjects with a non-managerial job or no job were more likely to have knee OA and knee symptoms, the difference was not statistically significant (see Table 3). The associations of education level and income level with knee pain and prevalent radiographic knee OA remained significant after adjustment. Males until elementary school had 2.13 times higher risk of having knee OA or 2.61 times higher risk of having knee pain than males until college or above. Females until elementary school had 2.67 times high risk of having knee OA or 2.69 times higher risk of having knee pain than females until college or above level of education. Males with lowest quartile of income had 1.57 times higher risk of having knee OA and 1.92 times higher risk of having knee pain than males with highest quartile of income. Females with lowest quartile of income had 1.51 times higher risk of having knee OA and 1.60 times higher risk of having knee pain than females with highest quartile of income. When we ran the model to compare education and income level, the trend of association in relation to knee pain and prevalent radiographic knee OA was more evident in education level.

**Table 3:** Odds ratios of radiographic knee OA and knee symptoms by component of SES.

	Male			Female		
	Knee OA	knee pain	Knee stiffness	Knee OA	knee pain	Knee stiffness
**Education**						
College	1	1	1	1	1	1
High school	0.98 (0.607,1.581)	1.326 (0.635,2.767)	1.309 (0.953,1.798)	1.359 (0.855,2.16)	1.456 (0.733,2.889)	0.869 (0.564,1.34)
Middle school	1.958 (1.208,3.173)	1.962 (1.023,3.76)	1.421 (0.982,2.057)	1.716 (1.071,2.75)	1.679 (0.85,3.317)	1.055 (0.677,1.644)
Elementary	2.13 (1.38,3.286)	2.606 (1.446,4.696)	1.463 (1.027,2.086)	2.669 (1.713,4.158)	2.689 (1.421,5.091)	1.112 (0.72,1.717)
*P for trend*	< .0001	< .0001	0.055	< .0001	< .0001	0.1154
**Income**						
Q4	1	1	1	1	1	1
Q3	1.029 (0.646,1.64)	0.726 (0.35,1.506)	1.145 (0.826,1.587)	0.961 (0.735,1.256)	1.09 (0.787,1.51)	1.175 (0.913,1.513)
Q2	1.278 (0.838,1.95)	1.566 (0.873,2.809)	0.985 (0.713,1.36)	1.13 (0.876,1.458)	1.146 (0.839,1.567)	1.068 (0.834,1.367)
Q1	1.567 (1.02,2.407)	1.918 (1.036,3.551)	1.123 (0.797,1.581)	1.505 (1.167,1.942)	1.596 (1.156,2.204)	1.342 (1.054,1.708)
*P for trend*	0.019	0.0037	0.7064	0.0002	0.0017	0.0419
**Occupation**						
Managerial	1	1	1	1	1	1
Non-managerial	1.014 (0.967,1.062)	1.049 (0.978,1.125)	1.158 (1.116,1.2)	1.101 (1.074,1.129)	1.081 (1.052,1.11)	1.182 (1.152,1.212)
No job	1.022 (1.006,1.037)	1.024 (1.001,1.047)	1.088 (1.073,1.104)	1.047 (1.036,1.058)	1.056 (1.043,1.07)	1.106 (1.095,1.118)
*P-value*	0.0673	0.1844	0.0003	0.702	0.8448	0.7568

Adjusted for age, BMI, education, and income or occupation.

OA, osteoarthritis; SES, socioeconomic status.

## Discussion

In this study, we examined the relationship of socioeconomic status and prevalence of knee symptoms and radiographic knee OA. We compared the effect of each determinant of SES on the parameters of knee symptoms and radiographic knee OA and demonstrated the most important socioeconomic factor related to knee OA. Although all three factors were closely related to prevalence of knee OA and knee symptoms, our study showed occupation to be the weakest and education level the strongest relationship component of SES to impact the risk of radiographic knee OA and knee pain.

It has been reported that low education, non-managerial occupation, and low-income status are related to knee pain and prevalence of knee OA. However, no previous study evaluated the most powerful determinant of SES for knee OA. Most studies focused on the effect of occupation on prevalence of knee OA^12–17^. Occupational activities, especially physically demanding activities, were confirmed to be related to symptomatic knee OA^9^. Certain occupations were shown to increase the risk for knee OA^10,16^. Similarly, our study showed that occupation is an important factor determining OA severity and pain. After adjusting for occupation, however, we demonstrated that education status remained more significant and showed greater risk in determining knee OA and knee symptoms. Those with low compensation had 1.5 times higher risk of having knee OA or knee pain compared with those with high compensation, however, those until elementary education had 2.6 times higher risk of having knee OA or knee pain compared with those until college or above education. This finding was true for both men and women, and it was consistent after adjusting for age and BMI. There was a similar finding in a prior study that found an independent association of educational attainment with knee OA, without a significant association between occupation and knee OA^4^.

The reason behind the strong relationship between education attainment and prevalence of knee OA and knee pain is unclear. There could be some unmeasured confounding factors that affected the results. It also should be noted that we classified the job status into 3 categories (managerial, non-managerial and no job), and this rather gross classification might not have been detailed enough to capture the relationship between knee OA and complicated job situations. In addition, the no job status included various situations, including housewives, temporary workers, and freelancers, so the category was heterogeneous. Also, the job status and income were surveyed as current status, so it did not reflect the prior job or income if the subject had been retired. These remain as limitation of the study. On the other hand, education can be the most stable surrogate marker that represents an individual’s SES from childhood. Indeed, among the three SES components, education is the only marker that does not change by disease status^3^, whereas occupation and income can change after an individual is diagnosed with a disease. Therefore, low education level might be a better marker of lower SES among the three components, demonstrating a more stable association with OA risk factors and knee pain.

Osteoarthritis, the most common form of arthritis, affects more than 15% of the global population and is a leading cause of disability^18^. In this study, we showed that all three components of SES were related to symptoms and prevalence of knee OA, but we also showed that less educated people were more likely to suffer from knee OA and pain. It is unclear how the low education status affects radiographic knee OA. It may affect the person’s lifestyle due to lack of knowledge about OA or the lifestyle itself due to low education status. These people are reported to be less likely to utilize medical and surgical managements and remain disabled with pain^19,20^. Also, all three factors of SES are not very modifiable and hard to intervene. However, education can be modifiable in a certain extent. In order to decrease the global health burden and disability caused by knee OA, we should try to intervene patients with low SES, especially the low educated people by giving them educational sessions about OA and offering treatment options. The strategies to lessen the future burden of OA should focus on earlier screening and improved provision of treatment options for those with less educated.

## Methods

### Study participants^21,22^

We conducted a cross-sectional study using data from the Fifth Korean National Health and Nutrition Examination Survey (KNHANES V) that were collected between 2010 and 2012. We previously published similar studies^21,22^ using KNHANES data. KNHANES is a nationally representative cross-sectional survey that has been conducted by the Korean Centers for Disease Control and Prevention since 1998. It is designed to assess the health and nutritional status of the non-institutionalized civilian population in Korea and is comprised of a health interview, a health examination, and a nutrition survey. We included male and female participants 50 years or older. This study used de-identified national survey data from KNHANES V, and all participants provided written informed consent for use of data for research purposes. This study was granted an exemption by Institutional Review Board of Seoul St. Mary’s Hospital (KC21ZASI0086).

### Variables

The patient characteristics obtained from the survey data were age, sex, BMI, waist circumference, and social history, which included current smoking, heavy drinking, residence setting (urban or rural), exercise, education status, income level, and occupation. Current smoking, heavy drinking, urban residence, exercise, and presence of a spouse were binary variables. Education status was categorized into 4 groups by the highest level of education achieved by the participant: elementary school, middle school, high school, and college. Income level was categorized into quartiles. Occupation was divided into 3 groups of managerial work, non-managerial work, and no job. Bilateral standing anteroposterior and lateral plain radiographs of the knees were obtained using SD3000 Synchro Stand (Accele Ray, Shinyoung Co., Seoul, South Korea), and they were monitored and evaluated by a group of radiographic technicians. The average score of the X-ray quality was 82.09 of 100. Two independent radiologists graded the degree of knee OA according to the Kellgren-Lawrence (KL) grade. The higher grade between the two was taken if a discrepancy was noted. However, if the discrepancy was equal or more than two grades, a third radiologist adjudicated to arrive at a consensus. After grading both knee joints, the higher grade was determined as the KL grade of each patient. The concordance rate of KL grades between the two radiologists was 94.76%. The inter-rater agreement, kappa coefficient, for the KL grading was 0.65. The radiologists had no knowledge about any participant’s knee symptoms^22^. Radiographic knee OA was defined as KL grade equal or greater than 2. Symptomatic knee OA was defined as presence of pain or stiffness among those with radiographic knee OA. Presence of knee pain was a dichotomous variable in which a person experienced or did not experience knee joint pain for more than 30 days over the previous 3 months. Presence of knee stiffness was indicated by morning knee stiffness for more than 30 days over the previous 3 months.

### Statistical analyses

The KNHANES survey used a stratified multistage cluster sampling design, which creates non-independence along with disproportionate sampling^23^. To avoid bias, we weighted the eligible participants according to the proper methods recommended by KNHANES to provide nationally representative prevalence estimates. Therefore, we used standard error rather than standard deviation. We compared groups with and without radiographic knee OA using the t-test, chi-square test, and analysis of variance according to type of variable. Due to the distinct difference by sex, analyses were performed separately for each sex. Analyses of covariance were used to examine the relationship between each component of SES and prevalence of knee OA or its symptoms. Multivariable logistic regression models were used to compare the effect of each component of SES on prevalence of radiographic knee OA and knee symptoms after adjusting for age, body mass index, and the other two SES components using SAS© version 9.3 (Cary, NC, USA).
